# Compact Electromagnetic Bandgap Structures for Notch Band in Ultra-Wideband Applications

**DOI:** 10.3390/s101109620

**Published:** 2010-11-01

**Authors:** Mihai Rotaru, Jan Sykulski

**Affiliations:** School of Electronics and Computer Science, University of Southampton, Southampton, SO17 1BJ, UK; E-Mails: mr@ecs.soton.ac.uk (M.R.); jks@soton.ac (J.S.); Tel.:+44-0-2380599613; Fax: +44-0-2380593709

**Keywords:** electromagnetic bandgap structures, EBG, UWB communication systems

## Abstract

This paper introduces a novel approach to create notch band filters in the front-end of ultra-wideband (UWB) communication systems based on electromagnetic bandgap (EBG) structures. The concept presented here can be implemented in any structure that has a microstrip in its configuration. The EBG structure is first analyzed using a full wave electromagnetic solver and then optimized to work at WLAN band (5.15–5.825 GHz). Two UWB passband filters are used to demonstrate the applicability and effectiveness of the novel EBG notch band feature. Simulation results are provided for two cases studied.

## 1. Introduction

There is growing interest in studying electromagnetic bandgap (EBG) structures for applications at microwave frequencies. New EBG designs have been used primarily to enhance the functionality of antennas [1], but other applications—such as filters and baluns—have also been explored [2]. Moreover, the EBG structures have features that can be used to reduce or suppress electromagnetic interferences (EMI) that occur in electronic systems leading to electromagnetic compatibility (EMC) issues [3]. The EBG structures suppress the propagation of surface waves over specific frequency bands that directly depend on the dimensions and types of materials used to fabricate the EBGs.

In this paper we focus on a slightly different application that uses only a unit cell of an EBG structure. Consider a notch band structure that can be used in ultra wideband (UWB) radio systems and be easily integrated with microstrip circuitry fabricated with printed circuit board (PCB) technology. Since the release in February 2002 of the 3.1–10.6 GHz band for commercial communication usage by the Federal Communication Commission (FCC), UWB has received a lot of attention. Unlike other existing wireless communication standards, which are narrowband, UWB has a very wide bandwidth of 7.5 GHz. However, the UWB emission power is limited to a maximum of −41.3 dBm/MHz, therefore it can co-exist with other narrow band services that occupy the same spectrum. One such service is the 802.11a WLAN that is located at 5.15–5.45 GHz and 5.725–5.825 GHz. Recent work has shown that the effect of the 802.11a interference on UWB can be harmful and—depending on the probability of signal overlap and the relative distance between the two transceivers—can cause significant signal degradation of the attainable throughput of the UWB system [4]. Hence it is very important to incorporate means to mitigate the effects of 802.11a in the UWB front end. Different types of structures for the physical layers and techniques for the MAC layers have been suggested recently. The previously proposed notch filter solutions are very specific to certain types of filters or antennas, therefore they cannot be easily integrated in a different design [5–7]. In this paper we propose a more general approach that can be implemented in any physical design that has at least a microstrip structure in its front end.

## 2. The EBG Structure

EBG structures are used for different applications and the most popular mushroom-like EBG was first introduced by Sievenpiper in 1999 [1]; its performance can be explained by a simple equivalent LC parallel resonant circuit. More recently the EBG structures have been used to suppress the noise propagating in parallel plate waveguide structures, such as the power planes of high speed electronic systems. The equivalent circuit used to describe the EBG behaviour in an open environment is somewhat different to the initial LC parallel resonant circuit used by Sievenpiper. Due to the EBG’s proximity to the two metal planes, the capacitances to the plane above and below the mushroom are much higher than those between the edges of the adjacent mushrooms and will therefore dominate the response of the EBG structure.

**Figure 1.**
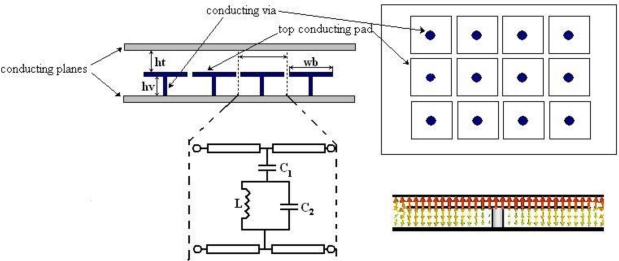
EBG embedded between two metal planes and its equivalent circuit.

In this configuration the EBG behaves like a stop band filter for the electromagnetic wave propagating in the parallel plate waveguide. The centre of the stop band frequency and the bandwidth are determined by *C_1_*, *C_2_* and *L*, where *C_1_* is the capacitance between the top conducting pad and the metal structure above, C_2_ is the capacitance between the pad and the bottom metal plane, and *L* is the inductance of the via connecting the bottom metal plane to the pad (Figure 1). *C_1_* and *C_2_* are determined by the size of the pad, the distance from the top and bottom planes and the dielectric material between the two planes. *L* is mostly influenced by the size of the connecting via (length, diameter) but also by its position with respect to the centre of the patch.

**Figure 2.**
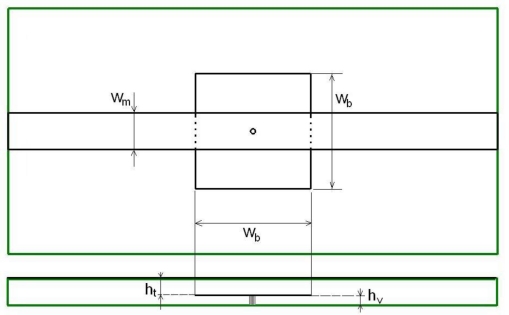
Mushroom EBG coupled to a microstrip line.

**Figure 3.**
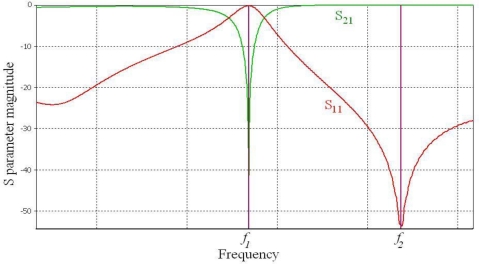
S11 and S21 magnitude variation with frequency (simulation results).

As the distance between the two parallel plates is much smaller than the size in the *xy* direction, it may be assumed that only a TEM mode is travelling in this waveguide. The equivalent circuit of an EBG unit shown in Figure 1 suggests that if an EBG unit cell is embedded within another wave guiding structure supporting a TEM mode there should be a similar band stop filter-like response. This was confirmed by Horii [5] but also through our simulation of a microstrip line run above an EBG unit cell (one mushroom—Figure 2). The structure has been simulated in CST Microwave [9]; the microstrip line has a width *w_m_* of 1.5mm and is routed on top of a 0.8mm thick FR4 substrate with *ε_r_* = 4.4. The bottom side of the substrate is covered by a continuous ground plane. The characteristic impedance of the transmission line was calculated to be 50 Ω. A typical result of the simulation in terms of the magnitudes of the scattering parameters is shown in Figure 3.

As before, the behaviour can be explained by the circuit in Figure 1. The return loss of this arrangement of a microstrip line above one mushroom (Figure 2) has a zero at frequency *f*_2_ and a pole at *f_1_* as shown in Figure 3, where *f*_1_ and *f_2_* are:

(1) $$f_{1}=\frac{1}{2\pi \sqrt{L(C_{1}+C_{2})}}\;f_{2}=\frac{1}{2\pi \sqrt{LC_{2}}}$$

Hence the stop band appears at a frequency *f_1_*.

The properties described above may be used to create a simple and efficient notch filter; however, the circuit parameters for this structure cannot be extracted directly from its geometry which makes the design of such a filter difficult. It has been suggested in [3] that a simple approximation—such as a parallel plate capacitance for the *C_1_* and *C_2_*—will suffice when the mushroom EBG is used between a parallel plate configuration. When only one cell is coupled to a transmission line, as in our proposed approach, the distribution of the fields around the structure changes significantly from the case when a full EBG matrix is embedded between two close metal planes. However, the structure of the equivalent circuit does not change. The difference is in the value and in the way in which the values of the equivalent circuit change with variation of the mushroom geometry. The equivalent circuit based on the circuit shown in Figure 1 can be extracted using the following approach.

The two equations presented earlier (1) may be re-written for example as follows

(2) $$C_{1}=\frac{1}{L}\frac{f_{2}^{2}-f_{1}^{2}}{{\left(2\pi f_{2}f_{1}\right)}^{2}}\;\text{and}\;C_{2}=\frac{1}{L{\left(2\pi f_{2}\right)}^{2}}$$

where two (out of three) circuit parameters are expressed in terms of the third one and the two resonant frequencies, which in turn may me assumed from a simulation or measured data. This procedure enables the equivalent circuit of Figure 1 to be established by fitting the frequency response in terms of the S-parameters to a set of S parameters obtained from a full wave simulation (Figure 3) or from measurements. As there is now only one circuit parameter to vary, it is relatively easy to obtain a good match. Once the best fit has been found the circuit parameters (*C_1_*, *C_2_* and *L*) are automatically available for a particular geometry. Any of the three parameters may be used as an unknown in the above simple procedure.

**Figure 4.**
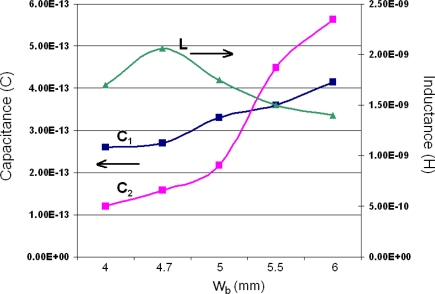
*L*, *C_1_* and *C_2_* variation with *w_b_* for a mushroom EBG coupled to a microstrip line.

Using the procedure described above several different designs of a mushroom EBG coupled to a microstrip line have been analyzed in an attempt to relate the geometry and the size of the EBG to its equivalent circuit parameters. The height of the via *h_v_* and its position were kept fixed and the width of the EBG *w_b_* was varied. The full wave simulations were run and the resulting S-parameters were imported into a circuit simulator in order for the equivalent circuits to be fitted to these results. As expected, when the size of the mushroom top was increased (by increasing *w_b_*), the resonant frequencies *f_1_* and *f*_2_ shifted to lower values. Moreover, the relative distance between these two frequencies dropped, thus reducing the bandwidth of the notch filter created by this combination. This behaviour was to be expected as when the size of the mushroom top is increased the capacitances *C_1_* and *C_2_* will increase. However, an interesting behaviour of the capacitance and inductance of this structure was observed when the values of the extracted equivalent circuits were analysed. The findings are summarized in Figure 4.

It is clear that *C_1_* and *C_2_* indeed increase as *w_b_* increases; however, *C_2_* starts to dominate for *w_b_* = 5.5 mm and above, a result which was less obvious to predict. It is also noted that the inductance *L* of the EBG increases initially but for values of *w_b_* above 4.7 mm starts to drop. Another observation worth mentioning is related to the actual values of *C_1_*, *C_2_* and *L*. Both capacitances have values below 1pF, whereas the inductance varies between 1.4 and 2 nH, hence the inductance of the structure has a much stronger influence on the values of *f_1_* and *f_2_*. It can also be observed from (1) that *L* and *C_2_* have a stronger effect over the bandwidth of the notch; when they increase the bandwidth is reduced. So for a narrow band notch filter higher values of *C_2_* and *L* are necessary.

The size of the EBG structure becomes critical when practical implementation aspects are taken into consideration. Unfortunately, in this case, the size of a mushroom EBG to be integrated into a realistic substrate, such as a FR4 board with a thickness of 0.8 mm, is quite large (4.7 × 4.7 mm) if, say, 5.4 GHz is chosen as the resonant frequency and the height of the via *h_v_* (Figure 2) is assumed to be 0.3 mm. But even for such a large element the 3 dB bandwidth of the notch is only about 15%, which is not really useful for an application such as UWB which requires a sharp cut-off frequency.

**Figure 5.**
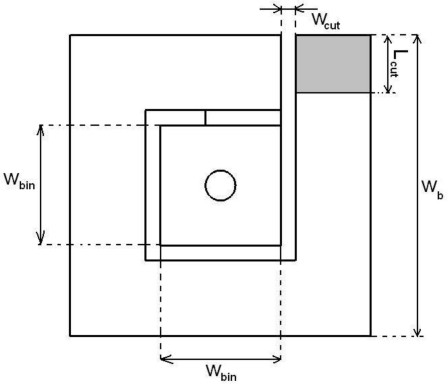
Top view of the new EBG.

To address the issues discussed above, and to make good use of the properties of a single mushroom EBG coupled to a microstrip line, the solution is proposed, where a modified top side mushroom cell as shown in Figure 5 is introduced.

A spiral cut (Figure 5) is inserted onto the top side of the mushroom, transforming the top side into a small planar inductor. Using this approach a 5.5-fold reduction in the area covered by the unit cell can be achieved and a much sharper cut-off of the resultant notch filter for the same FR4 substrate and same height of the via as before. The notch middle frequency was assumed to be 5.4 GHz as in the previous case. The size of the cut *w_cut_* was taken as 0.1 mm, the inner width *w_bin_* = 1.2 mm and the outer *w_b_* = 2 mm.

This structure should behave in a very similar manner to the unit cell mushroom EBG, hence the same equivalent circuit as before could be used; however, a more complex equivalent circuit might be needed if higher frequency effects were to be considered. For this work the main interest was around 5.5 GHz, therefore only the simpler circuit was investigated.

**Figure 6.**
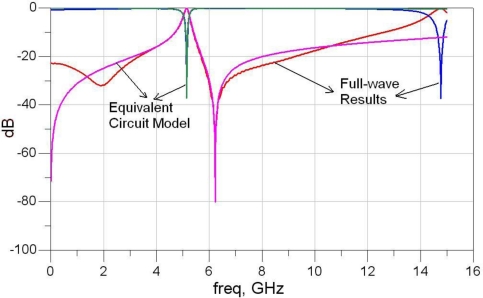
S-parameters for the coupled inductor-mushroom EBG—comparison between full-wave and equivalent circuit results.

Figure 6 shows a comparison between the magnitudes of the S parameters obtained from simulation and the same parameters calculated using the extracted equivalent circuit. A good match is observed within the frequency range of interest, around *f_1_* and *f_2_*; however, for higher frequencies the simple circuit model is not accurate.

**Figure 7.**
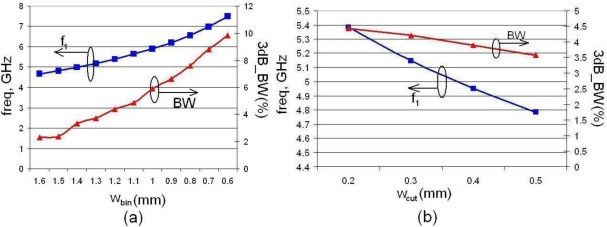
Variation of resonant frequency *f_1_* and 3dB bandwidth with *w_bin_* **(a)**, and *w_cut_* **(b)**.

**Figure 8.**
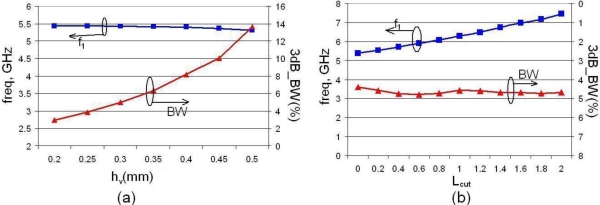
Variation of resonant frequency *f_1_* and 3dB bandwidth with *h_v_* **(a)**, and *L_cut_* **(b)**.

In addition to the two parameters used in the design of a mushroom configuration, namely its width and the via height, the new structure has two more parameters that can be used to tune the structure for the frequency and bandwidth required. The two extra parameters are *w_bin_* and *w_cut_* (Figure 5). The former is the width of the inner portion of the structure; if its size is varied the resonant frequency may be changed quite dramatically but its total footprint remains unaffected, unlike in the mushroom unit. Another important parameter that is influenced by *w_bin_* is the bandwidth of the notch. As *w_bin_* is reduced, *f_1_* and *f_2_* move towards higher frequencies and the bandwidth becomes larger [Figure 7(a)]. For the purpose of fine tuning the latter parameter, *w_cut_*, may be used. For a fixed *w_bin_* increasing *w_cut_* will reduce the resonant frequencies and will also slightly reduce the bandwidth, as shown in Figure 7(b). If a change in the resonant frequency is desired but without a change in the bandwidth, an extra design parameter may be introduced in the form of *L_cut_*, which is a length that could be removed from the beginning of the planar inductor top (as shown in Figure 5); as *L_cut_* increases the resonant frequency is shifted to a higher value without changing the bandwidth [Figure 8(b)]. Finally, if the bandwidth is to be modified at a fixed frequency, the via height *h_v_* may be used as a design parameter; when *h_v_* is increased the bandwidth increases and vice versa [Figure 8(a)].

## 3. UWB Filters with Embedded EBG Element

Using the proposed methodology, small footprint structures may be designed with notch band characteristics that can be incorporated into existing designs without substantial and costly modifications. To illustrate this, two existent band pass filters for UWB applications [6,7] were modified to incorporate a notch band feature based on the inductor EBG and designed for the WLAN. The applicability and usefulness of the new structure is further evident as the two filters chosen are very different in structure and are made of very different substrates. The following results were obtained through full wave simulation using CST Microwave Studio [9].

**Figure 9.**
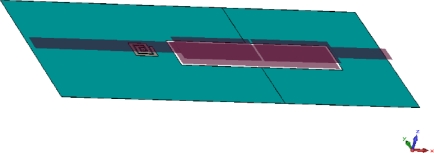
A 3D view of a broadside coupled microstrip-coplanar waveguide UWB bandpass filter (filter 1) with embedded EBG unit.

The first UWB filter studied here is based on the broadside coupling between a microstrip and a coplanar waveguide (CPW) [6]. The CPW is on the ground of the microstrip, while the two microstrip lines on the top surface are separated by a small gap. This UWB filter structure is assumed to be on a dielectric substrate with *ε_r_* = 2.17 and a thickness of 0.508 mm. The metal thickness its 18 μm. The metal elements of this filter are illustrated in Figure 9, all the dielectric components (substrate) are hidden such the embedded EBG is visible. The filter has a size of about 2 × 5 cm with a 5 mm length for the input and output microstrip lines. Using the methodology explained in Section II, an inductor based mushroom EBG with a 2 × 2 mm footprint and height of *h_v_* = 0.2 mm was designed embedded within the filter substrate coupled to the input microstrip line. The simulation results showed a very sharp notch centred at 5.68 GHz with a rejection of loss of about 30 dB and with a 3 dB bandwidth of about 5.6% (Figure 11).

**Figure 10.**
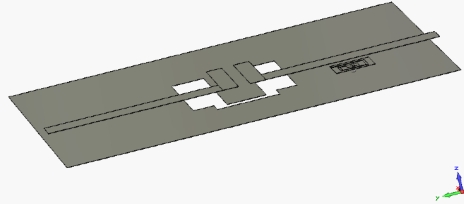
A 3D view of the L-shaped coupled microstrip UWB bandpass filter (filter 2) with embedded EBG unit.

**Figure 11.**
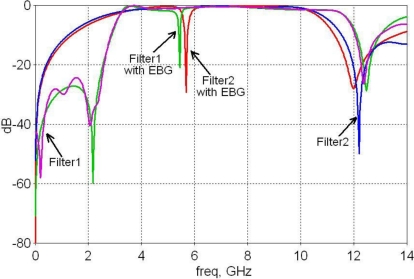
Magnitude of the return loss for UWB filters with and without an embedded EBG unit.

The second UWB filter [7] has two coupled L-shaped microstrips on the top layer and a stepped impedance resonator (SIR) on a defected-ground structure (DSG) on the bottom layer. Figure 10 presents the metal structure of the UWB filter with the EBG embedded within the substrate. Once again the dielectric substrate is not shown in figure 10 for clarity. This filter is designed for a FR4 substrate with *ε_r_* = 4.4 and a thickness of 0.8mm. Its total size is 1.5 × 1 cm. The mushroom inductor EBG structure embedded within this substrate and coupled to one of the L-shaped microstrips from the top layer had a footprint of 2 × 2 mm and *h_v_* = 0.3 mm. The simulation of the filter with the embedded EBG showed a sharp notch centred at 5.43 GHz with the rejection loss of about 21 dB and 3.75% 3 dB bandwidth (Figure 11).

## 4. Conclusions

In this paper a novel notch band filter based on an inductor mushroom EBG element coupled to a microstrip line has been investigated theoretically exploiting field modelling using electromagnetic simulation. A design methodology for the structure has been proposed and implemented, to demonstrate its usefulness in the context of an ultra-wideband bandpass filter application, as a notch filter for a 802.11a WLAN. The new structure is very simple and can be implemented in any design that has microstrip lines; it has the advantage of a small footprint and extensive tune-ability.
